# Delay-Aware and Link-Quality-Aware Geographical Routing Protocol for UANET via Dueling Deep Q-Network

**DOI:** 10.3390/s23063024

**Published:** 2023-03-10

**Authors:** Yanan Zhang, Hongbing Qiu

**Affiliations:** School of Information and Communications, Guilin University of Electronic Technology, Guilin 541004, China

**Keywords:** unmanned aerial vehicles ad hoc network (UANET), geographical routing, dueling deep Q-network, link quality, waiting time

## Abstract

In an unmanned aerial vehicles ad hoc network (UANET), UAVs communicate with each other to accomplish intricate tasks collaboratively and cooperatively. However, the high mobility of UAVs, the variable link quality, and heavy traffic loads can lead to difficulties in finding an optimal communication path. We proposed a delay-aware and link-quality-aware geographical routing protocol for a UANET via the dueling deep Q-network (DLGR-2DQ) to address these problems. Firstly, the link quality was not only related to the physical layer metric, the signal-to-noise ratio, which was influenced by path loss and Doppler shifts, but also the expected transmission count of the data link layer. In addition, we also considered the total waiting time of packets in the candidate forwarding node in order to decrease the end-to-end delay. Then, we modeled the packet-forwarding process as a Markov decision process. We crafted an appropriate reward function that utilized the penalty value for each additional hop, total waiting time, and link quality to accelerate the learning of the dueling DQN algorithm. Finally, the simulation results illustrated that our proposed routing protocol outperformed others in terms of the packet delivery ratio and the average end-to-end delay.

## 1. Introduction

The development of unmanned aerial vehicles (UAVs) has grown substantially over the past decade, and UAVs are extensively used in military, commercial, and civilian applications, such as in emergency communications [1], meteorological surveillance [2], agriculture and forestry monitoring and assessment [3], mineral exploration [4], and task offloading for mobile edge computing [5], among others. In these applications, multiple UAVs form an ad hoc network (UANET), which is a self-organizing, distributed network architecture that is more flexible and scalable than centralized network architectures. The nodes can communicate with others to complete specific tasks efficiently in a UANET. The communication between UAVs is a challenge, however, due to frequent topology changes and link quality fluctuations as a result of the high mobility of UAVs, as well as congestion due to heavy network traffic.

To accommodate the characteristics of a UANET, many protocols have improved upon conventional routing protocols. For example, in [6], the authors presented the improved OLSR based on the original optimized link-state routing (OLSR) protocol [7] to control the high overhead. In [8], the authors made use of the ad hoc on-demand distance vector (AODV), and Reynolds’ boids were used for connectivity and path maintenance while a packet was being forwarded. These proactive and reactive routing protocols relied on network topology information and needed to establish and maintain a routing table, which is not appropriate for bandwidth-limited and highly dynamic UANETs.

Therefore, a classical geolocation-routing protocol (GPSR) [9] was suggested that used geolocation information to assist in routing decisions. The routing protocol based on a geographical location was more suitable for highly dynamic UANETs because it was more scalable and had a lower overhead that only requiring the neighboring location information. The strategy of GPSR greedily selects the next-hop node, which provides the nearest distance to the destination node. It is essentially a shortest-route algorithm. If none of the neighboring nodes are closer to the destination node than itself, the target node forwards the packet by peripheral forwarding, leading to more overhead and delay. The authors of [10,11,12] presented a solution to address the routing-void problem. Although conventional routing protocols were simple and easily applied to UANETs, the aforementioned protocols were not intelligent and lacked autonomous routing decisions and adaptability, making them challenging to modify for a changeable UAV network environment with complex tasks.

As artificial intelligence has advanced, reinforcement learning (RL) has become an important class of algorithms in artificial intelligence, and applications based on reinforcement learning have achieved effective results in edge computing [13], resource allocation [14], routing decisions [15,16,17], and so forth. Reinforcement learning is a methodological framework for learning, predicting, and making decisions. If a problem can be described or transformed into a sequential decision problem, then RL can be used to solve it. The advantage of RL-based routing protocols is that they have a long-term vision, i.e., long-term return, which allows them to select better decisions than conventional routing protocols, which only consider the immediate reward. The authors of [18] proposed a new Q-learning-based routing protocol for flying ad hoc networks, which considered two-hop neighboring nodes and adaptively selected the next-hop node based on the network characteristics, such as location, delay, velocity, and resident energy level. However, Q-routing-based routing protocols have the following limitations: (1) they cannot be adapted to large-scale network scenarios because learning a large-scale Q-value table is time-consuming and convergence is not guaranteed; (2) the scalability of the protocols is insufficient, therefore, when new nodes join or leave the network, the state space and action space changes, requiring the entire network to be retrained; (3) they are unable to handle continuous state spaces. Therefore, the authors of [19] proposed a routing system based on a deep Q-network (QNGPSR). However, it did not consider the increase in delays and packet losses caused by heavy-load scenarios. In [20], the authors proposed a traffic-aware routing protocol to adapt congestion routing based on a Q-network (TQNGPSR). In [21], the researchers considered the link stability and predicted the resident energy when selecting the next-hop node. However, these routing protocols did not consider the link quality and total waiting time for packets in the neighboring node’s buffer before propagating over the channel.

Motivated by the above research, we propose a routing protocol that utilizes a dueling double-deep Q-network that considers the link quality and total waiting time. The main contributions of this study are the following:

- First, we evaluated the link quality that combined the physical layer metric, the signal-to-noise ratio (SNR), and the data link layer metric, the expected transmission count (ETX). The long distances between nodes and the nodes’ high mobility could lead to a significant path loss and Doppler shift, respectively. However, the SNR considered the path loss and Doppler shift. The ETX metric was used to reflect the link quality degradation caused by bursty link anomalies or malicious link attacks.
- Secondly, as opposed to the method described in [20], which used the number of packets in a candidate forwarding node’s buffer as a penalty factor, we considered the total waiting time of packets in the candidate forwarding node’s buffer before they were propagated over the communication channel, which was more accurate because the SNR influenced the transmission rate. The transmission rate affected the transmission delay.
- Lastly, we proposed a dueling DQN-based routing protocol that trained nodes to make routing decisions intelligently and automatically. The packet-forwarding process was modeled as a Markov decision process. Furthermore, the reward function was designed to accelerate the learning process. The experiments proved our proposed routing protocol resulted in better network performance.

The remainder of our paper is organized according to six sections. In Section 2, we describe the related work. Section 3 presents the system model. The proposed routing protocol based on a dueling DQN is detailed in Section 4. Section 5 describes the parameter settings and analyzes the experimental results of different routing protocols. Section 6 provides the conclusions drawn based on this study.

## 2. Related Work

Routing protocols for UANETs have been widely investigated in review articles [22,23,24]. The methods can be roughly divided into two categories. Conventional routing protocols and reinforcement-learning-based routing protocols. Conventional routing protocols include topology-based and geographical-location-based routing protocols.

An example of topology-based routing protocols is the reactive routing protocol. Many routing protocols have been improved based on the original AODV. For example, the authors of [25] optimized the AODV protocol by considering resident energy and relative movement. The authors of [26] proposed an energy-aware, cross-layer AODV routing protocol to reduce link breakage and improve network performance. In [27], the authors proposed an HN-AODV routing algorithm. When the sequence number of the route request or route reply packet was equal to the sequence number of the recorded route request or route reply packet stored in a node, the number of neighbors and the hop count along the path were considered to decrease the end-to-end (E2E) delay during the path discovery process.

Another example of topology-based routing protocols is proactive routing, which is a table-driven routing similar to reactive routing. The most typical proactive routing protocol is OLSR. Many improvements of OLSR have considered the characteristics of UANETs. In [28], the performance of the OLSR protocol was improved by adjusting the parameters and decreasing the holding times. In [29], the authors presented the novel and effective OLSR+, which calculated the link lifetime according to distance, link quality, relative velocity, and movement direction, and selected multipoint relays based on fuzzy logic based on residual energy, link lifetime, and the number of neighbors. Furthermore, the included route energy and route lifetime into the topology control message. In [30], the authors used multibeam directional antenna (MBDA) technology to forward a packet in different directions without radio frequency interference, which ensured that OLSR had a low probability of detection. In addition, a multipath-enhanced OLSR based on MADA was proposed, and a social network concept was applied to select the multipoint relays with small broadcast overheads.

Geographical-based routing protocols do not need routing tables; instead, they require the maintenance of neighboring tables, which makes them suitable for highly dynamic networks. Since GPSR is capable of mitigating routing voids, the authors of [31] proposed a recovery strategy to resolve this problem. The recovery strategy involved three methods. In the first, when a routing void occurred, the current forwarding node waited and attempted to resend the packet. The second method forwarded packets to the farthest neighboring node. The third method forwarded packets to the node with the fastest movements, which was determined by predicting the distance information. The novel routing protocol in [32] used the location estimation of the destination to deliver packets in an appropriate direction while considering congestion information to forward a packet for delay-tolerant and energy-limited UAV networks. The authors of [33] presented an adaptive “hello” mechanism and a greedy forwarding mechanism according to the relative motion of the nodes. Overall, the conventional routing protocols have been relatively simple and easy to implement, but they lack the ability to self-adapt or to employ intelligent and autonomous decision-making.

In recent years, multiagent autonomous, intelligent decision-making systems based on artificial intelligence have become a research hot spot. Many researchers have utilized RL to solve routing decision problems because RL-based routing protocols can adapt to highly dynamic topology changes in a UANET [34]. In [35], the authors proposed the Q-Fuzzy approach. Fuzzy logic was used to select the next-hop node by considering link-related parameters. After discovering the destination node, Q-learning calculated the Q-value of the path-related parameters to evaluate the efficiency of the entire path. Q-learning enabled the fuzzy system to conserve energy and ensured good communication performance. Being highly dynamical with a variable link quality, a couple of studies [36,37] proposed improved Q-learning-based routing protocols with adaptive learning rates. Meanwhile, others improved the exploration parameters of Q-learning to adapt to different mobile scenarios. For example, in [38], the simulated annealing (SA) optimization was used to self-adaptively control the exploration rate through the declining temperature rate. The authors of [39] utilized Q-learning to perform a joint optimization of E2E delay and energy consumption and adaptively adjusted the Q-learning parameters based on the link conditions. In addition, an exploration and exploitation mechanism was introduced to evaluate potential optimal routing paths.

Since the aforementioned routing protocols based on Q-tables were not scalable and could not be adapted for large-scale networks, researchers proposed deep Q-network-based routing protocols. In [40], the authors used a DQN to understand the relationship between the best routing decisions and the localized information of the forwarding nodes.Furthermore, they utilized a deep value network (DVN) with a feedback mechanism to leverage the system dynamics in order to further increase the adaptability and efficiency of the DQN routing. The authors of [41] assigned the UAVs as relay nodes to compensate for broken links, adopted a DQN model to select the optimal decision in order to adapt to a dynamic topology and variable link conditions, and a priority queue model was designed to improve the quality-of-service (QoS) performance. In [42], the researchers proposed an adaptive and reliable routing protocol using a DQN that considered the link status when making routing decisions. The authors of [43] exploited a DQN to design a vertical routing protocol that considered residual energy and mobility rates.

## 3. System Model

In this section, we describe the UANET architecture and introduce the link-quality-estimation metric as well as the metric of total waiting time of packets in a candidate forwarding node before the packets propagate over the communication channel.

### 3.1. Network Architecture

We implemented a UANET, which consisted of *N* homogeneous nodes and communication links. We assumed the packet transmission power of each node was the same, so the communication radius *R* was the same. Each node could communicate with its neighboring nodes within the communication radius. Due to the limitation of the wireless communication range of the UAVs, some UAVs were unable to communicate with others directly and required assistance from the relay nodes to forward packets. If node *i* could directly communicate with the neighboring node *j*, we assumed the link $L_{i,j}\left(i\neq j\right)$ existed. Each node had a full-duplex transceiver, a unique identity document (ID), and a global positioning system (GPS) to provide the location, speed, and direction information. In addition, each node could acquire neighbor information by receiving hello packets periodically.

### 3.2. Link Quality

Due to the rapid movements of the UAVs, the network topology changed frequently, making wireless links unreliable. Other factors also influenced the quality of the received signal, such as signal fading, attenuation, and Doppler shifts. These factors led to packet loss. Therefore, the accurate evaluation of wireless link quality was vital for designing routing protocols that could guarantee network performance.

The link quality estimators were divided into hardware-based and software-based estimators. The hardware-based link quality estimators were obtained from the physical layer module. The software-based link quality estimators were obtained by calculating the expected transmission count from the data link layer. In order to obtain a more comprehensive and accurate evaluation of the link quality, we considered the SNR and the expected transmission count in our calculations. As mentioned earlier, the path losses and Doppler shifts in the UANET were key factors that influenced the SNR.

#### 3.2.1. Path Loss

In this paper, we assumed that all UAVs flew at the same altitude. Transmitting and receiving UAVs that could communicate directly without any obstacles affecting their communication quality used line-of-sight (LOS) propagation. In free spaces, the path loss of transmitting and receiving nodes was caused by the transmission power’s radiation and dispersion and the channel’s transmission characteristics. Furthermore, the distance between the receiver and transmitter was proportional to the path loss, according to the Friis transmission equation.The path loss of $L_{i,j}$ was expressed by the following:

(1) $${\mathrm{Los}}_{i,j}\left(d_{i,j}\right)=10\mathrm{lg}{\left(\frac{4\pi }{c}\right)}^{2}+\alpha 10\mathrm{lg}d_{i,j}+20\mathrm{lg}f$$

where α=2.05 is the path loss exponent [44]; $d_{i,j}=\sqrt{\left(x_{i}^{2}-x_{j}^{2}\right)+\left(y_{i}^{2}-y_{j}^{2}\right)}$ is the Euclidean distance between node *i* and its neighboring node *j*; and $\left(x_{i},y_{i}\right)$ and $\left(x_{j},y_{j}\right)$ are the location coordinates of nodes *i* and *j*, respectively. The variable *f* is the carrier frequency, and *c* is electromagnetic wave speed, which is approximately equal to the speed of light.

The above equation calculated the dB value of the path loss, and we converted it to a formal absolute value, $PL\left(d_{i,j}\right)=10^{\frac{{\mathrm{Los}}_{i,j}\left(d_{i,j}\right)}{10}}$.

#### 3.2.2. The Effect of the Doppler Shift

In a UANET, the Doppler shift is a significant phenomenon in which the received signal frequency is not the same as the transmitted signal frequency when there has been relative motion between the transmitter and the receiver. The magnitude of the Doppler shift is proportional to the magnitude of the relative velocity. If the difference between the received and the transmitted signal frequencies is high, it significantly influences the communication quality.

When node *i* communicated with its neighboring node *j*, the movement of receiving node *j* led to a distance difference Δl of the received signal, and the value of the phase difference variation of the received signal could then be expressed as the following:

(2) $$\Delta \varphi =\frac{2\pi \Delta l}{\lambda }=\frac{2\pi v_{j}\Delta t}{\lambda }\cos\theta$$

where θ is the angle between the direction of the radio wave incidence and the direction of movement of node *j*; $v_{j}$ is the speed of movement; and λ=c/f is the wavelength of the transmission signal. Based on this, the value of the frequency change, i.e., the Doppler shift $\Delta f_{i,j}$ could be obtained as follows:

(3) $$\Delta f_{i,j}=\frac{\Delta \varphi }{2\pi \cdot \Delta t}=\frac{v_{j}}{\lambda }\cdot \cos\theta$$

Based on Equation (3), the Doppler shift was not only related to the relative movement speeds of the transmitter and receiver but also the angle between the direction of the radio wave incidence and the direction of the movement. If node *j* traveled toward node *i*, the $\Delta f_{i,j}$ was positive, i.e., the received signal frequency increased. If node *j* traveled away from node *i*, the $\Delta f_{i,j}$ was negative. The received frequency decreased.

Doppler shifts degraded the performance of the received signal. If the Doppler shift $\Delta f_{i,j}$ was equal to the symbol rate, the signal power would exceed the band limit of the filter. To solve this problem, a simple solution was to widen the filter bandwidth of the receiving node, so the SNR loss caused by the Doppler frequency shift was calculated as follows:

(4) $$df_{i,j}=10\cdot \log\left(\frac{2\cdot \Delta f_{i,j}+R_{d}}{R_{d}}\right)=10\cdot \log\left(\frac{2\cdot \Delta f_{i,j}}{R_{d}}+1\right)$$

where $R_{d}$ is the symbol rate.

The result of Equation (4) is in dB, so we converted it to a formal absolute value as follows:

(5) $$DF_{i,j}=10^{\frac{df_{i,j}}{10}}=\frac{2\cdot \Delta f_{i,j}}{R_{d}}+1$$

#### 3.2.3. SNR Calculation

According to the above considerations, the path loss and Doppler shift were considered as the primary factors of the SNR. However, shadowing fading was also considered in the channel state information. The shadowing effect is caused by obstacles between the transmitter and the receiver, i.e., non-LOS (NLOS) transmissions, which degrade the signal power through absorption, reflection, scattering, and diffraction; in severe cases, it can even block the signal entirely. The SNR between node *i* and its neighboring node *j* was expressed as follows:

(6) $$SNR_{i,j}=\frac{P_{i}{\left|h_{i,j}\right|}^{2}}{N_{0}\cdot PL_{i,j}\cdot DF_{i,j}}$$

where $P_{i}$ is the hello transmission power of node *i*, and $h_{i,j}$ is the fading coefficient of link $L_{i,j}$ following the Nakagami-m channel, which included the effect of shadowing fading. The expression $N_{0}∼N\left(0,\sigma^{2}\right)$ is the power of an additive white Gaussian noise (AWGN) with a noise variance $\sigma^{2}$, $PL_{i,j}$ is the absolute form of the path loss for link $L_{i,j}$, and $DF_{i,j}$ is the absolute form of the loss value of the Doppler shift.

#### 3.2.4. ETX Metric

In addition to the influence of the wireless communication channel characteristics, bursty link anomalies or malicious link attacks by other devices could also lead to packet loss. Therefore, relying only on the SNR to measure link quality was not feasible. As a result, we used the expected transmission count (ETX) as another link quality evaluation metric to assist routing decisions, which was critical to decrease packet retransmission and loss on poor links. The ETX measured the number of expected packet transmissions that could be successfully received at the receiver node. We counted the successful probability of receiving hello packets by both end nodes of the bidirectional link. The ETX of the link between node *i* and node *j* was expressed by the following:

(7) $$ETX_{i,j}=\frac{1}{d_{f}^{i,j}\times d_{r}^{i,j}}$$

where $d_{f}^{i,j}$ is the successful delivery probability of node *j* receiving a hello packet from node *i* during the last *w* seconds, and $d_{r}^{i,j}$ is the successful delivery probability of node *i* receiving a hello packet from node *j* during the last *w* seconds.

#### 3.2.5. Link Quality Evaluation

Therefore, by integrating the expected transmission count and the SNR, the link quality between node *i* and its neighboring node *j* could be defined as follows:

(8) $$LQ_{i,j}=\mu SNR_{i,j}^{*}-\nu ETX_{i,j}^{*}$$

where $LQ_{i,j}^{*}$ and $ETX_{i,j}^{*}$ are the normalized values of the link quality and the expected transmission count of link $L_{i,j}$, respectively. μ and ν are the weights of the expected transmission count and SNR, respectively, and 0<μ,ν<1, because the ETX was as small as possible, so the negative sign was added before the weight.

### 3.3. Total Waiting Time of Packet

**Figure 1:**
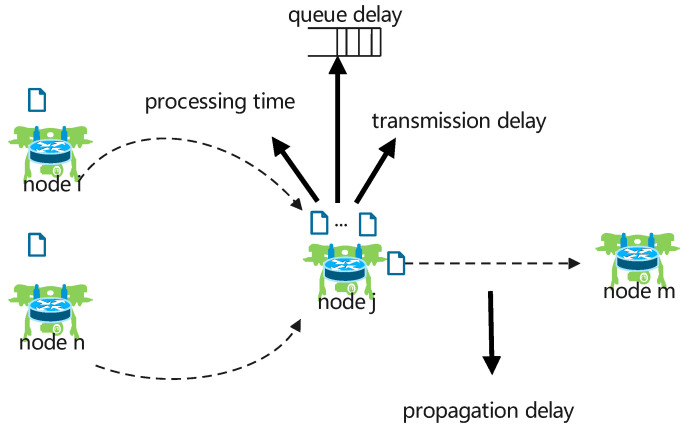
Composition of the point-to-point delay between two nodes.

In a UANET, when a node sends a packet to its neighboring node, the waiting time of the unprocessed packets in the neighboring nodes increase the end-to-end delays and even increase the packet losses during heavy traffic conditions. Therefore, the current forwarding node considered the waiting time of packets in neighboring nodes to select an appropriate node while still meeting the minimum delay requirements. Point-to-point delays consist of the queue time of packets in the node’s buffer, the processing delay, the transmission delay, and the propagation delay, as shown in Figure 1. Because we only wanted to estimate the waiting time of packets in the neighboring node, we ignored the packets’ propagation delay over the communication channel.

Each node was expected to have the same buffer size. The scheduling mechanism of packets in the buffer was based on first-in, first-out (FIFO). When current forwarding node *i* forwarded a data packet to next-hop node *j*, the total waiting time of packets in node *j* at current time *t* could be calculated by the following:

(9) $$T_{j}^{\mathrm{wait}\;}\left(t\right)=\left\{\begin{array}{cc} \sum_{k=2}^{m}\left(\frac{P_{\mathrm{size}}}{R_{i,j}}+T_{\mathrm{proc}\;}^{k}+T_{\mathrm{queue}\;}^{k}\right) & k\geq 2\\ \frac{P_{\mathrm{size}\;}}{R_{i,j}}+T_{\mathrm{proc}\;}^{k} & k=1 \end{array}\right.$$

where $P_{\mathrm{size}}$ is the size of a data packet, and $T_{\mathrm{proc}}^{k}$ is the processing delay of the *k*th packet in the buffer. $T_{\mathrm{queue}}$ is the queue delay of the *k*th packet in the buffer, $R_{i,j}=W\cdot \log_{2}\left(1+SNR_{i,j}\right)$ is the packet transmission rate during the communication link $L_{i,j}$, *W* is the bandwidth allocation for the communication link between node *i* and node *j*, and *m* is the total number of packets in the buffer of node *j*. If there was only one packet in the buffer of the candidate forwarding node, i.e., k=1, the packet could be transmitted immediately. Otherwise, the packet would queue and wait to be scheduled for transmission.

## 4. Dueling DQN-Based Routing Protocol

In this section, we describe the model of the packet-forwarding process as a Markov decision process. Then, the dueling DQN algorithm structure and decision-learning process are described in detail. Finally, we describe the routing protocol based on our proposed dueling DQN algorithm.

### 4.1. Markov Decision Process

In order to use the RL algorithm to solve the routing problem, the packet-forwarding process should be modeled as a Markov decision process (MDP). An MDP consists of four tuples {S,A,R,P}, where *S* is the state space, *A* the is the action space, *R* is the reward function, and *P* is the state transfer probability. According to our UAV-routing scenario, the specific definitions of these tuples were the following:

(1) State space: $S=\left\{s_{1},s_{2},\ldots ,s_{N}\right\}$, where $s_{i}=\left\{d_{i,j},d_{j,d},d_{i_{2}^{n},d},d_{\mathrm{sum}\;},LQ_{i,j},DIR_{i,d},T_{j}^{\mathrm{wait}\;}\right.\left.|j\in Nbr(i)\right\}$, and $d_{i,j}$ is the distance between current forwarding node *i* and its candidate forwarding node *j*. In addition, $d_{j,d}$ is the distance between candidate forwarding node *j* and destination node *d*. We used the expression of neighboring topology information ${NF}_{x}=\left(d_{1},d_{2},\cdots ,d_{8}\right)$ to predict the location of the two-hop neighboring nodes of the current forwarding node in different regions [19].

The predicted location of the two-hop neighboring nodes was expressed as the following: $L_{2}\left(n,i\right)=l_{i}+d_{j}\left(\cos\frac{(1+2j)\pi }{8},\sin\frac{(1+2j)\pi }{8}\right)$, where $d_{j}$ indicates the farthest distance between the current forwarding node and its neighboring node in the *j*th region, and $l_{i}$ is the location of node *i*. The feature $d_{i_{2}^{n},d}$ is the minimum distance between the predicted two-hop neighboring nodes and the destination node in the *n* regions. Then, $d_{\mathrm{sum}}=\sum_{i=1}^{8}\min\left(\left|l_{i}l_{d}\right|-\left|l_{j}\left(x,n\right)l_{d}\right|,0\right)$, if $d_{\mathrm{sum}}$ was smaller, which indicated a larger probability of mitigating the routing void, where $l_{d}$ and $l_{d}$ are the location of destination node *d* and node *j*, respectively, $LQ_{i,j}$ is the link quality of the link $L_{i,j}$, and $T_{j}^{\mathrm{wait}}$ is the total waiting time of packets in node *j* at the current time. The feature $DIR_{i,d}$ was used to learn to avoid selecting a candidate forwarding node that was traveling far away from the destination node, which decreased the probability of packet transmission success. The specific expressions of $DIR_{i,d}$ were expressed as follows:

(10) $${\mathrm{DIR}}_{i,d}=\frac{\vec{V_{j}}\cdot \vec{l_{i}l_{d}}}{∥\vec{V_{j}}∥∥\vec{l_{i}l_{d}}∥}$$

where $\vec{V_{j}}$ is the velocity vector of candidate forwarding node *j*, $l_{l}$ is the location of last-hop forwarding node *l*, and $\vec{l_{l}l_{d}}$ is the vector of node *i* to destination node *d*. If $0<{\mathrm{DIR}}_{i,d}<1$, then candidate forwarding node *j* was traveling closer to node *d*. If $-1<{\mathrm{DIR}}_{i,d}<0$, then *j* was traveling farther away from node *d*.

All these features were designed so the agents could quickly learn the optimal policy. Additionally, different features had different units and scales. We normalized these dimensional features into dimensionless features for data processing convenience and accelerating the Q-network convergence. In our study, we adopted the min–max normalization method.

(2) Action space: where $A_{i}=\left\{a_{1},a_{2},\ldots ,a_{n}\right\}$ is the set of neighboring nodes within the communication range of current forwarding node *i*, and *n* is the total number of neighbor agents. The action was selected when the current forwarding node needed to forward a packet.

(3) Transition probability: $P:S_{i}\times a_{i}\times S_{j}\to \left[0,1\right]$ defined the probability that the current state would transition to the next state after taking an action.

(4) Reward function: $S_{i}\times A_{i}\to R$ was a critical component. A well-designed reward function could ensure the algorithm would converge faster while also improving network performance. Therefore, the reward function of the current node forwarding a packet to the next hop was designed as follows:

(11) $$Reward_{i,j}=\left\{\begin{array}{cc} -1+\varphi T_{j}^{wait*}+\delta \left(1-LQ_{i,j}^{*}\right) & \;\mathrm{if}\;\mathrm{node}\;j\;\mathrm{is}\;\mathrm{not}\;\mathrm{destination}\;\\ 100 & \;\mathrm{otherwise}\; \end{array}\right.$$

where −1 indicates that for each additional hop, the reward function is reduced by 1, δ is the weight of the link quality between nodes *i* and *j*, φ is the total waiting delay of packets in candidate forwarding node *j*, δ,φ<0. $T_{j}^{wait*}$ and $LQ_{i,j}^{*}$ are the normalized values of the total waiting time of the packet in node *j* and the link quality of link $L_{i,j}$, respectively. If candidate forwarding node *j* was the destination, we issued 100 rewards immediately when choosing node *j* as the next-hop forwarding node.

### 4.2. Routing-Decision-Learning Based on Dueling DQN

After modeling the packet-forwarding process as an MDP, we used a dueling DQN to learn routing decisions. The purpose of a reinforcement learning algorithm is to ensure agents learn an optimal policy $\pi^{*}\left(a|s\right)$. There are two methods employed to learn the optimal policy in RL algorithms. One is by learning the policy function directly, and the other is by learning the state function $V^{*}=\max V^{\pi }\left(s\right)$ or action function $Q^{*}=\max Q^{\pi }\left(s,a\right)$, indirectly. The state function $V_{k}^{\pi }\left(s\right)$ is the expectation of the sum of all rewards from current state *s* when forwarding the *k*th packet to final state *d* with the policy π, which is expressed by the following:

(12) $$V_{k}^{\pi }\left(s\right)=E^{\pi }\left\{\sum_{t=0}^{\infty }\gamma^{t}r_{k}\left(t+1\right)\right\}$$

where γ∈[0,1] is the discount factor, which is used to determine the compromise between immediate and future rewards. According to the Bellman equation, the action function $Q_{k}^{\pi }\left(s,a\right)$ can be expressed by the following:

(13) $$Q_{k}^{\pi }\left(s,a\right)=r_{k}\left(s,a\right)+\gamma E_{s^{\prime }∼p}\left[V_{k}^{\pi }\left(s^{\prime }\right)\right]$$

**Figure 2:**
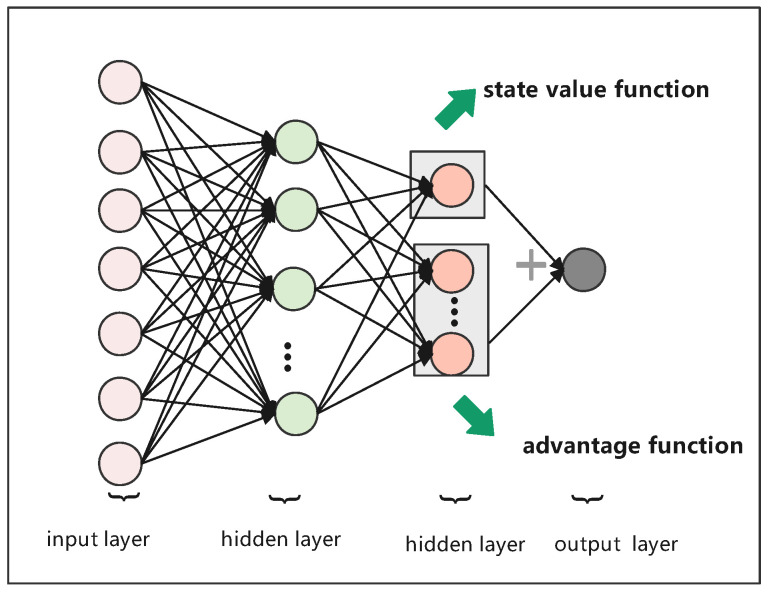
The neural network structure based on a dueling DQN.

As opposed to [19,20], we adopted a DQN to learn routing decisions. The Q-value was output by the neural network to evaluate the quality of the action. However, it could be inaccurate, because the Q(s,a) were related to both state and action, respectively. The degree of correlation between them was not the same. Therefore, in our paper, we utilized a deep dueling Q-network (dueling DQN) algorithm [45] to learn the routing decisions by optimizing the structure of the neural network. Instead of using the original paper’s convolutional neural network (CNN) network structure, we used a four-layer neural network to simplify the algorithm. Whether a CNN was used did not affect the dueling DQN algorithm in our scenario. The simple four-layer neural network had an input layer, two hidden layers, and an output layer, which is depicted in Figure 2.

For dueling DQN, the Q-value was divided into two parts. The first part was only related to state *s*, which had no relationship with specific action *a*. We referred to this as the state function V(s;w,α), which was scalar. The second part was related to both state *s* and action *a*, which we referred to as the advantage function A(s,a;w,β), which was a vector. Each value of the vector corresponded to one action. Then, the Q-value could be represented as follows:

(14) Q(s,a;w,α,β)=V(s;w,α)+A(s,a;w,β)

where *w* is the network parameters vector of the common part, α is the network parameter vector of the state function, and β is the network parameter vector of the advantage function. However, there was an unidentifiable problem, i.e., given a Q-value, it was impossible to obtain a unique state function and advantage function. To overcome this problem, the advantage function had to be processed by mean centering. Then, the Q-value could be rewritten, as follows:

(15) $$Q\left(s,a;w,\alpha ,\beta \right)=V\left(s;w,\alpha \right)+\left(A\left(s,a;w,\alpha \right)-\frac{1}{A}\sum_{a^{\prime }\in A}A\left(s,a^{\prime };w,\beta \right)\right)$$

There were two Q-networks in the designed dueling DQN algorithm structure: one was the current Q-network Q(s,a;w,α,β), and the other was the target Q-network $Q^{\prime }\left(s,a,w^{\prime };\alpha^{\prime },\beta^{\prime }\right)$, where w,α,β are the parameter vectors of the current Q-network, and $w^{\prime },\alpha^{\prime },\beta^{\prime }$ are the parameter vectors of the target Q-network. The objective function of the Q-network was to minimize the loss function $L\left(w_{t},\alpha_{t},\beta_{t}\right)$. The expression of the loss function was the following:

(16) $$L\left(w_{t},\alpha_{t},\beta_{t}\right)=E\left[{\left(Q_{\mathrm{target}\;}-Q\left(s_{t},a_{t};w_{t},\alpha_{t},\beta_{t}\right)\right)}^{2}\right]$$

where $Q_{\mathrm{target}}=E\left[R_{t+1}+\gamma Q^{\prime }\left(s_{t+1},a_{t+1};w^{\prime };\alpha^{\prime },\beta^{\prime }\right)\right]$, and the discount factor γ is a predetermined hyperparameter. We set it as 0.9, based on previous studies. Next, we used the adaptive moment estimation (Adam) to optimize the gradient of the loss function. The gradient of the loss function was expressed by the following:

(17) $$\nabla_{w_{t},\alpha_{t},\beta_{t}}L\left(\theta_{t}\right)=E\left[\left(Q_{\mathrm{target}\;}-Q(s,a;w_{t},\alpha_{t},\beta_{t}\right)\nabla_{w_{t},\alpha_{t},\beta_{t}}Q\left(s_{t},a_{t};w_{t},\alpha_{t},\beta_{t}\right)\right]$$

We utilized a soft update instead of a hard copy for updating the parameters of the target Q-network in order to conserve memory space and stabilize the learning process. The soft update was calculated by the following:

(18) $$\left\{\begin{array}{c} \alpha^{\prime }=\left(1-\tau \right)\alpha^{\prime }+\tau \alpha \\ \omega^{\prime }=\left(1-\tau \right)\omega^{\prime }+\tau \omega \\ \beta^{\prime }=\left(1-\tau \right)\beta^{\prime }+\tau \beta \end{array}\right.$$

where τ∈[0,1] denotes the compromise between the weights of the target Q-network and current Q-network. Although soft updates cost more time, it increased the learning stability.

In the training phase, for the sake of balancing exploration and exploitation, we proposed an improved upper-confidence-bound (IUCB) strategy to select the action, which was expressed as follows:

(19) $$a_{i}=\left\{\underset{a\in A_{i}}{\arg\max}\left\{Q_{i}\left(s_{i},a_{i}\right)+\sqrt{\frac{\ln N_{s_{i}}}{N_{s_{i},a}}}\right\}\right.$$

where $N_{s_{i}}$ denotes the number of states $s_{i}$ that were visited within a fixed period of time, and $N_{s_{i},a}$ denotes the number of times to select the *k*th largest Q-value of candidate forwarding nodes in state $s_{i}$ within this fixed period of time. The meaning of the parameters $N_{s_{i}}$ and $N_{s_{i},a}$ were different from the original UCB policy. They were designed according to our scenario, as compared to [19,20], in which they adopted a ε-greedy strategy for the action. Although the ε-greedy strategy was easy to achieve, the IUCB performed better than the ε-greedy strategy. The IUCB not only considered the Q-value but also the number of times to select the *k*th largest Q-value of the candidate forwarding nodes. Therefore, it could fully explore the action that had been left unexplored at the beginning, and then it tended to exploit that action. After constant iterations, the algorithm achieved convergence.

In the testing phase, we used a softmax strategy to select the next-hop forwarding node, that is, we selected the next-hop node based on the probability instead of selecting the next-hop node with the maximum Q-value. The benefit of softmax was that it avoided localized optimums and split the traffic, which was expressed by the following:

(20) $$P\left(a\right)=\frac{\exp\left(Q\left(s_{i},a\right)/\tau \right)}{\sum_{a_{i}\in A_{i}}\exp\left(Q\left(s_{i},a_{i}\right)/\tau \right)}$$

where $A_{i}$ is the set of actions of node *i*, and τ is the temperature parameter; according to our experience, we set τ=0.05. If τ was large, the probability distribution of different candidate forwarding nodes had few differences, resulting in a larger loss. If τ was small, the probability distribution of different candidate forwarding nodes had more significant differences, resulting in a smaller loss.

### 4.3. The Proposed Routing Protocol

**Figure 3:**
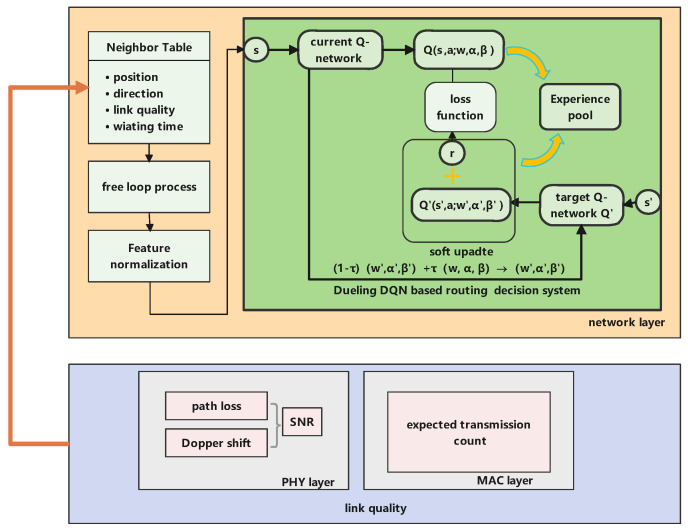
The framework for our proposed routing protocol.

The framework of our proposed routing protocol is shown in Figure 3. The core module was a dueling DQN-based geographical-routing decision system in the network layer. This module was responsible for learning each node’s Q-value according to the input state information. The data link layer and physical layers were used to acquire the ETX and SNR metrics, which could be used for estimating the link quality. At the same time, each node needed to extract additional state information from the established neighboring table and execute a free route loop operation.

According to the designed routing protocol architecture, the routing process was divided into two phases: the first phase was the neighboring table establishment and maintenance, and the second phase was the routing decision.

#### 4.3.1. Neighboring Table Establishment and Maintenance

**Figure 4:**
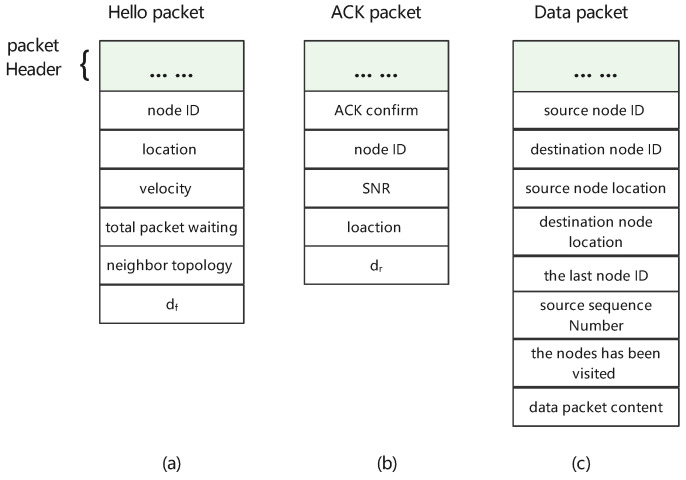
The format of the packets. (**a**) Hello packet. (**b**) ACK packet. (**c**) Data packet.

Each node broadcast hello packets periodically at predefined intervals *t* to exchange useful state information that contained its geographical location, velocity, probability value ($d_{r}$), packet total waiting time, and node ID. Figure 4a displays the format of a hello packet. When a node received a hello packet from its neighboring node, it established entry in its neighboring table NT and recorded related information from the hello packet.

After the node received the hello packet, the receiving node replied with an ACK packet to the current forwarding node to confirm the link had been established. Furthermore, an ACK packet included node ID and location, ACK confirmation, and the SNR and probability values ($d_{f}$). Furthermore, the neighboring table also recorded useful information to assist in routing decisions. This is shown in Figure 4b.

Additional information needed to be recorded in the neighboring table during the process of data-packet forwarding to assist in routing decisions. The data-packet format is shown in Figure 4c. The data packets included node ID and the location of the destination and source nodes, the nodes that the packet had already visited (HVN), which was used to avoid routing loops, a unique sequence number to decide the freshness of the packet, the last node’s ID, and the data packet content.

#### 4.3.2. Routing Decisions

After the neighboring table was established, when a source node sent a data packet to the destination node, the routing decision process based on the dueling DQN algorithm was triggered. With the assistance of relay-node forwarding, the data packets finally reached the destination node.

When current forwarding node *i* needed to forward a packet, the process of the routing decisions was the following. First, current forwarding node *i* had to determine the number of its optional actions $A_{i}^{\mathrm{opt}}=A_{i}-HVN$. If $A_{i}^{\mathrm{opt}}$ = *∅*, current node *i* discarded the packet. If $A_{i}^{\mathrm{opt}}\neq \emptyset$, it proceeded to the next step. Second, current forwarding node *i* extracted the feature information $F_{x}=\left\{l_{i},l_{l},l_{d},l_{x},LQ_{i,x},T_{x}^{\mathrm{wait}},NF_{x}\right\}$ from the neighboring table and received the data packet (where $l_{i}$ is the location of current forwarding node *i*, $l_{l}$ is the location of last-hop forwarding node *l*, $l_{d}$ is the location of destination node *d*, $l_{x}$ is the location of neighboring node *x*, $LQ_{i,x}$ is the link quality of link $L_{i,x}$, $T_{x}^{wait}$ is the total waiting time of packets in the buffer of neighboring node *x*, and ${NF}_{x}$ is the neighboring topology information). According to these features, the state information of node *i* was $s_{i}=\left\{d_{i,x},d_{x,d\;},d_{i_{2}^{n},d},d_{\mathrm{sum}\;},LQ_{i,x},DIR_{i,d},T_{x}^{\mathrm{wait}}\right\}$. For a detailed definition of $s_{i}$, please refer to Section 4.1 (1). Third, we fed the normalized state $s_{i}$ into the dueling DQN network. Finally, current node *i* selected the next-hop node according to the Q-value output by the Q-network. If the Q-network was in the training phase, the strategy of the next-hop node selected depended on expression (19). The optimal decision was learned through iterative learning until the Q-network converged. In the testing phase, each node selected the next-hop node depending on the softmax function (20). The pseudocode of the routing decision process is presented in Algorithm 1.
**Algorithm 1** Delay-aware and link-quality-aware geographical routing forwarding protocol for UANET via dueling deep Q-network at current forwarding node *i***Input:** $L_{i}$: is the location of the current forwarding node *i*;
  *p*: is the data packet to be forwarded;
  $NT_{i}$: is the neighboring topology information;
**Output:** Select the next-hop node according to the Q-value;
1:Obtain a set of neighboring nodes $A_{i}$ by $NT_{i}$;2:Calculate the optional neighboring set $A_{i}^{\mathrm{opt}}$ that excludes nodes that the packet has visited (HVN)3:**if** $A_{i}^{\mathrm{opt}}$ = *∅* **then**4:   $a^{*}=-1$ and packet loss5:**else**6:   **for** *x* in $A_{i}^{\mathrm{opt}}$ **do**7:     Extract feature information $F_{x}=\left\{l_{i},l_{l},l_{d},l_{x},LQ_{i,x},T_{x}^{\mathrm{wait}},NF_{x}\right\}$ and calculate state feature $s_{i}=\left\{d_{i,x},d_{x,d\;},d_{i_{2}^{n},d},d_{\mathrm{sum}\;},LQ_{i,x},DIR_{i,d},T_{x}^{\mathrm{wait}\;}\right\}$8:     Input $s_{i}$ into our RL model and output $Q_{i}$9:     **if** the Q-network is in training phase **then**10:        Select next step according to (19);11:        Store $e_{t}=\left(s_{t},a_{t},r_{t},s_{t+1}\right)$ into experience set $d_{t}=\left\{e_{1},\cdots ,e_{t}\right\}$;12:        Update Q-network parameters ω,α,β and $\omega^{\prime },\alpha^{\prime },\beta^{\prime }$13:     **else**14:        State select next step using (20)15:     **end if**16:   **end for**17:**end if**


## 5. Simulation Results and Analysis

In this section, our proposed routing protocol was implemented, verified, and analyzed by comparing it with GPSR [9], QNGPSR [19], and TQNGPSR [20]. We tested the network performance under different speeds, packet-sending rates, and node densities to comprehensively prove the superiority of our algorithm.

The simulation experiments of all the protocols were conducted based on the Python coding language. All the UAVs were moving in a random direction model. The UAVs were distributed in a rectangular area of 2 km × 2 km. The communication radius was 350 m. Hello packets were broadcast periodically at fixed intervals of 0.5 s. The maximum speed of the UAVs ranged from 5 to 25 m/s. The input features were processed by the min–max normalization method, and SELU was used as the activation function. In the training process, Adam was used as the optimizer, and the learning rate was set to 0.001. The minibatch gradient descent was used to update the network parameters, and the batch size was 32. The neuron network was 7 ×16×8×1. The main parameters of the simulation experiment are shown in Table 1.

The convergence rate, the average end-to-end (E2E) delay, and the packet–delivery ratio (PDR) were used as evaluation metrics to compare the network performance of the routing protocols. The specific definitions of these evaluation metrics were as follows.

- Convergence rate: The convergence rate was the limit of the ratio of two consecutive errors. In our scenario, the convergence rate was evaluated by training 100 maps, according to the training results, to determine whether the algorithm converged and to analyze the convergence speed and the network performance at convergence.
- Average end-to-end delay: The average E2E delay was measured by calculating the average delay of the data packets from the source node to the destination node.
- Packet–delivery ratio: The PDR was measured by calculating the ratio of successfully received data packets of the destination node to the number of total transmission packets of the source node.

### 5.1. Convergence Analysis

In the training phase, we trained 100 different maps that were scraped from flight trajectory data around the Paris Charles de Gaulle Airport in November 2017 from https://www.flightradar24.com/(accessed on 5 October 2021). We extracted the location information of 100 nodes from each map as the initial locations. Then, we compared the convergence rates of TQNGPSR, QNGPSR, and DLGR-2DQ. We used the packet–delivery ratio as a determinant of convergence rate.

**Figure 5:**
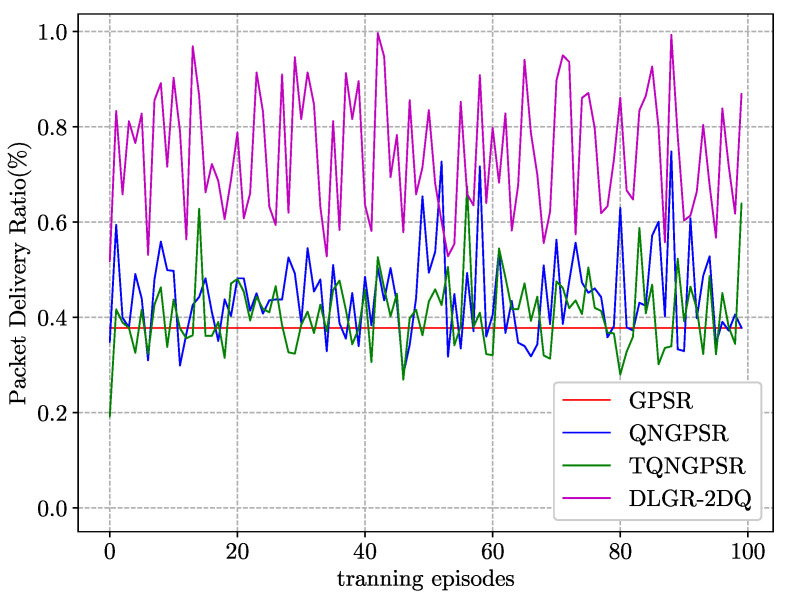
Convergence performance comparison of PDR among TQNGPSR, QNGPSR, and DLGR-2DQ during the training phase.

Figure 5 shows the PDR of the learning curve of all the protocols on different maps with continuous iterations. As shown, the GPSR did not need to be trained, so it was a straight line. The nodes were stationary in the training phase for all the protocols. That was because they were hard to converge when the nodes were mobile. The PDR of our proposed routing protocol was best compared with other protocols, and the PDR of the DLGR-2DQ fluctuated around 0.7. This was attributed to the link quality and total waiting time of packets in candidate forwarding nodes, which could significantly improve the packet–delivery ratio under a relatively heavy traffic load.

### 5.2. Comparison of Exploration Strategy in the Training Phase

**Figure 6:**
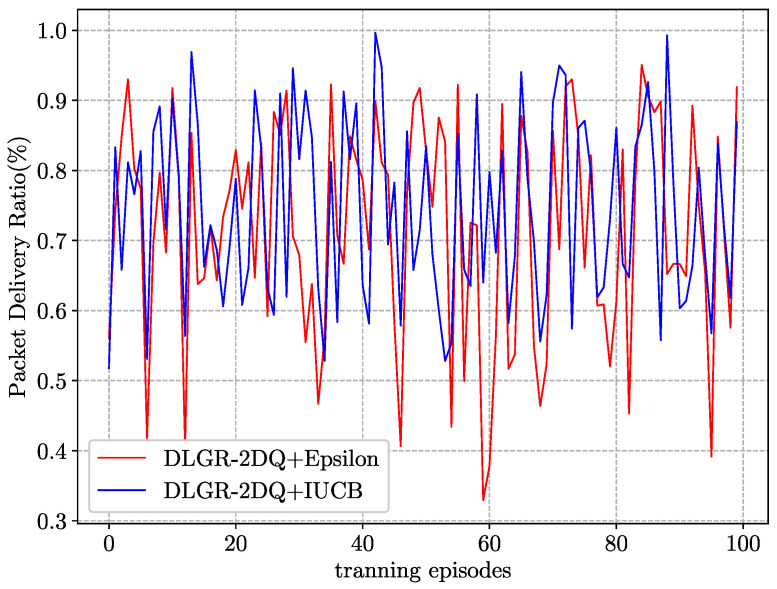
Convergence performance comparison of different exploration strategies during the training phase.

Figure 6 depicts the convergence performance of our proposed routing protocol that adopted different exploration strategies during the training phase. We compared the convergence rate of the ε-greedy and the IUCB exploration strategy. As shown, the variance in the PDR for the IUCB exploration strategy was approximately 30% smaller than that for the ε-greedy strategy. When exploration had been executed for a period of time, all the actions were nearly explored; then, the IUCB policy tended to greedily select the actions with the maximum Q-value, so the performance improved. This was in contrast to the ε-greedy strategy, which always randomly selected the next-hop node with a certain probability, resulting in a performance degradation.

### 5.3. The Impact of Network Density

**Figure 7:**
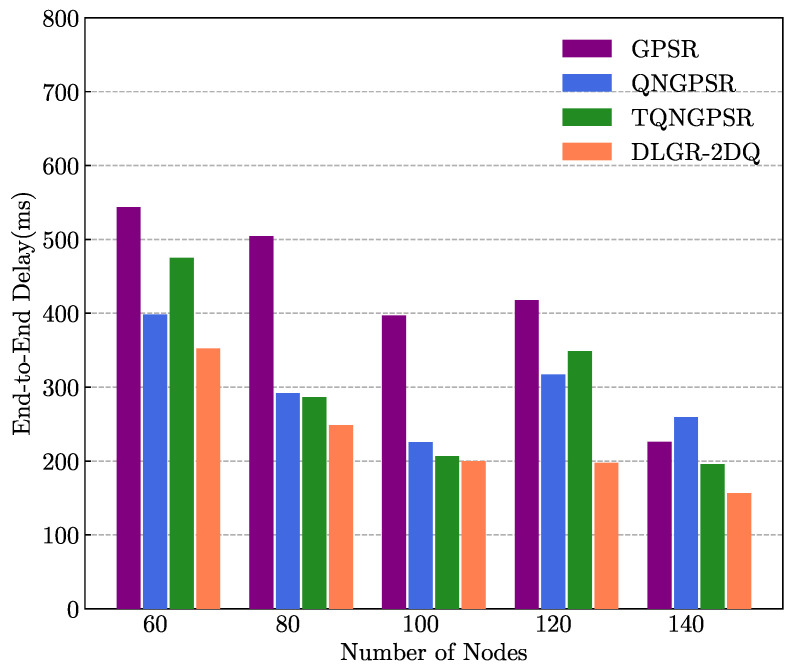
The effects of network density on E2E delay.

**Figure 8:**
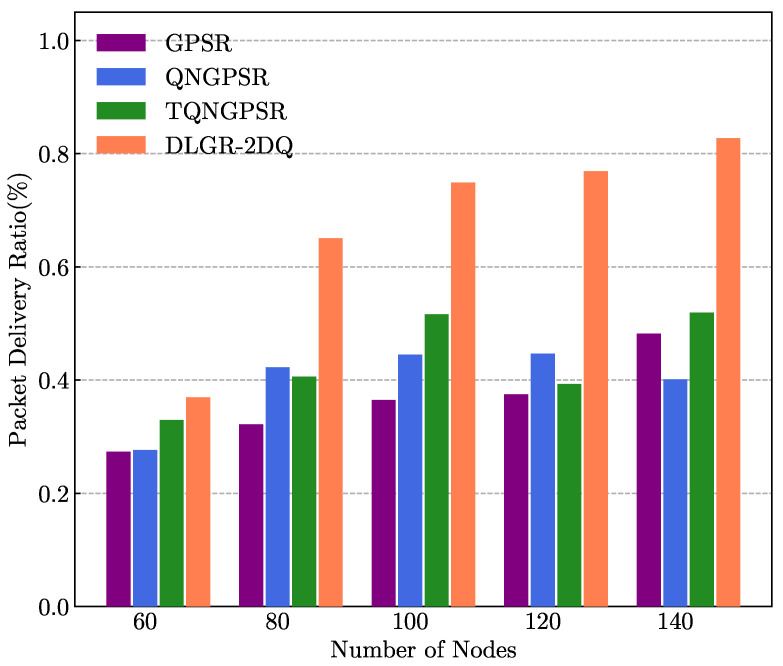
The effects of network density on PDR.

In the testing phase, for the purpose of evaluating and analyzing the network performance in different network densities for the GPSR, QNGPSR, TQNGPSR, and DLGR-2DQ protocols, we tested different numbers of nodes in the UANET that varied in the range of 40∼140. Figure 7 and Figure 8 present the average E2E delays and PDRs under different network densities, respectively. There were 100 nodes in the network. The minimum speed of the nodes was 3 m/s, and the maximum speed of the nodes was 10 m/s. The packet-sending rate was 5 Hz.

As shown in Figure 7, the E2E delay of DLGR-2DQ outperformed the other three routing protocols. Since DLGR-2DQ considered the total waiting time in the reward function, it required that each node learn to avoid selecting a congested neighboring node. As the number of the nodes increased, the delays in DLGR-2DQ gradually decreased. That was because the network density increased as the number of the nodes increased, so the probability of reaching the destination node with the minimum number of hops increased.

As shown in Figure 8, as the network density increased, more nodes assisted in forwarding packets to the destination node, so the PDR of all the routing protocols increased. The PDR of DLGR-2DQ was also significantly better than the other routing protocols, because DLGR-2DQ considered the link quality as a factor of the reward function and thus avoided forwarding packets on a poor-quality link between the current forwarding node and a candidate forwarding node.

### 5.4. The Impact of Maximum Speed

The purpose of the simulation experiment in this part was to investigate and analyze the impact of maximum speed on the network performance of these protocols. The number of nodes was 100 in the UANET. The minimum speed of the nodes was 3 m/s. The maximum speed change of the nodes ranged from 5 m/s to 25 m/s. The packet-sending rate was 5 Hz.

**Figure 9:**
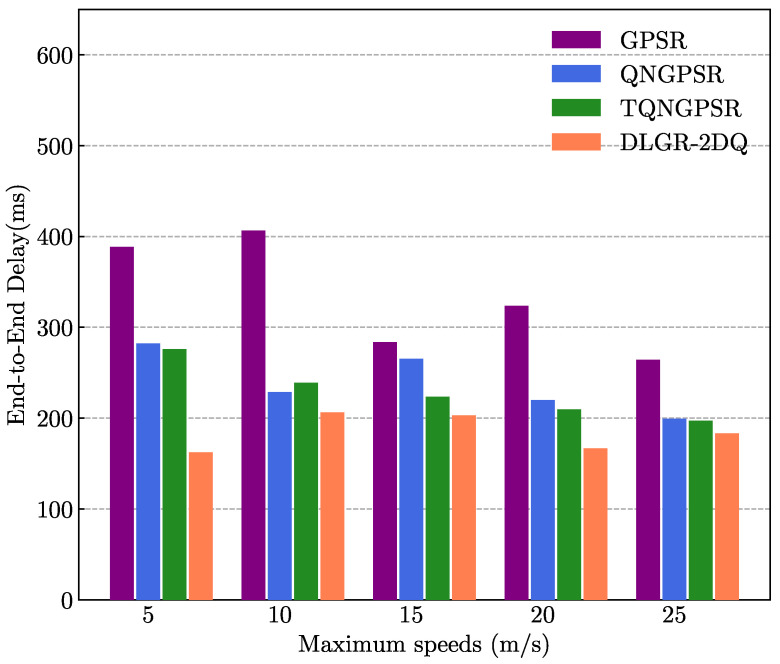
The effects of maximum speed on E2E delays.

Figure 9 presents the relationship between the maximum speed and the average E2E delay. As shown, the average E2E of the DLGR-2DQ was lower than that of the other protocols. The reason was that the DLGR-2DQ considered the total waiting time. When the buffer of a neighboring node of the current forwarding node was under a heavy load, or the packet transmission rate was very small due to a poor SNR, this increased transmission time in the neighboring node. Eventually, the E2E delays increased.

**Figure 10:**
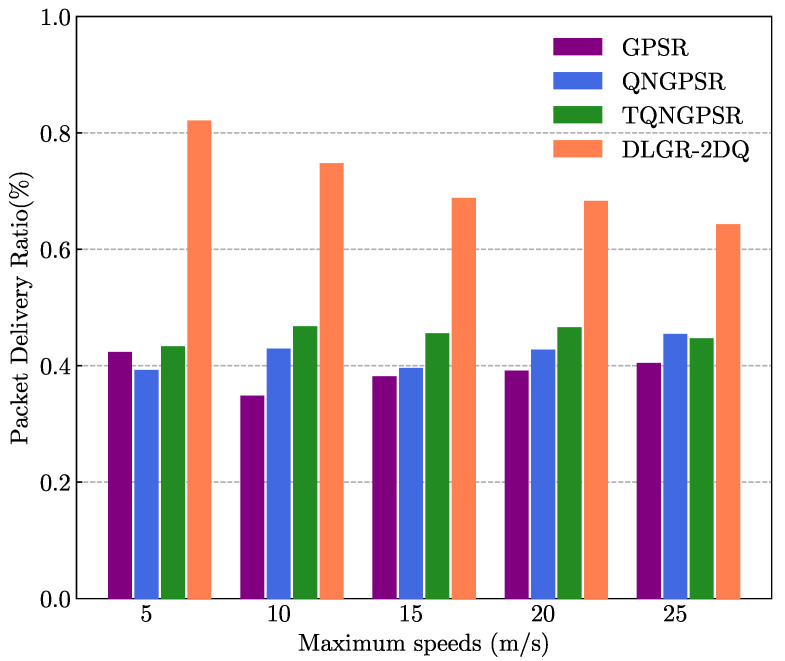
The effects of maximum speed on PDR.

Figure 10 depicts the impacts of maximum speed on the PDR. It was evident that DLGR-2DQ performed better than the other protocols at different maximum speeds. As the nodes’ maximum speed increased, the trends of the PDR for DLGR-2DQ decreased. This was because the larger the speed difference between adjacent nodes, the greater the Doppler shift, the worse the link quality, and the lower the packet delivery rate. The GPSR, QNGPSR, and TQNGPSR did not consider link quality, so the packet loss increased. TQNGPSR was slightly better than QNGPSR. That was because TQNGPSR considered the number of packets in the buffer of neighboring nodes and added the relevant penalty term to the Q-value.

### 5.5. The Impact of Packet Sending Rate

The target of the simulation experiment conducted in this part was to analyze the effects of the packet-sending rate on the E2E delays and PDRs. The packet-sending rate changed between 1 and 5 Hz. A total of 100 nodes were in the UAV network. The minimum and maximum speeds were 3 m/s and 10 m/s, respectively.

**Figure 11:**
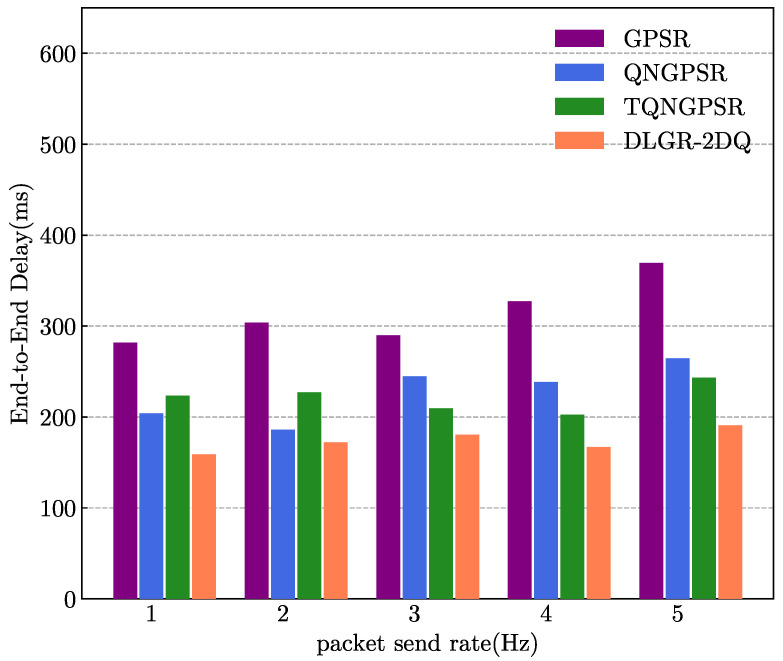
The effects of packet-sending rates on E2E delays.

Figure 11 illustrates the results of the average E2E delays versus the packet-sending rates. As seen from Figure 11, DLGR-2DQ had the best performance, compared to GPSR, QNGPSR, and TQNGPSR. This was because the total waiting time of the packets in each candidate forwarding node was considered before they were propagated over the channel. As the packet rate increased, the delay of DLGR-2DQ remained relatively stable, i.e., with a small variance.

**Figure 12:**
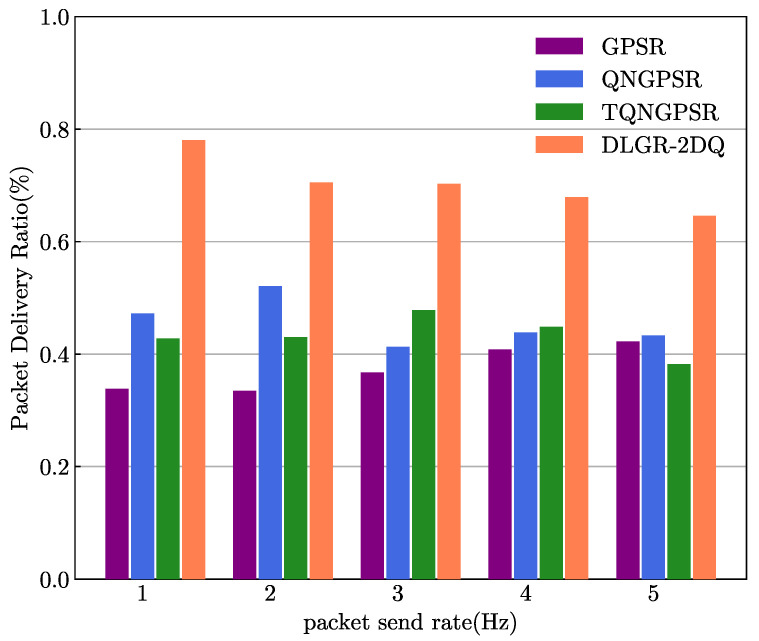
The effects of packet-sending rate on PDR.

Figure 12 illustrates the results of the PDR versus different packet-sending rates. The PDR of DLGR-2DQ was the largest, due to the total waiting time of packets in a candidate forwarding node being considered in our proposed routing protocol. DLGR-2DQ avoided forwarding packets to congested nodes and considered the link quality, so the packet loss was the smallest, compared to that of the other three routing protocols. It was clear that with the increase in the packet-sending rate, the PDR of DLGR-2DQ decreased.

## 6. Conclusions

In this paper, we proposed an intelligent, autonomous, distributed geographical routing protocol using a dueling DQN algorithm in a UANET, in order to mitigate the Doppler shifts caused by the high mobility of UAVs, lost paths due to long distances between two adjacent nodes, and the packet losses due to link anomalies, as these significantly impact network performance. Therefore, our proposed routing protocol used the SNR and the ETX as link quality estimation metrics, and we considered a packet’s total waiting time at candidate forwarding nodes before packets were propagated over the channel. We conducted experiments to analyze and compare our proposed routing protocol DLGR-2DQ with other routing protocols (i.e., GPSR, QNGPSR, and TQNGPSR). The simulation results demonstrated that DLGR-2DQ performed better than the other routing protocols in terms of convergence rates, PDRs, and average E2E delays, under different parameter variables, showing that our proposed routing protocol was robust.

## Figures and Tables

**Table 1:** Simulation parameters.

Parameter	Value
Movable region	2 km × 2 km
The number of UAVs	40∼140
Communication radius	350 m
Hello interval	0.5s
Packet sending rate	1 Hz∼5 Hz
Hello packet transmission power	1 W
Data packet size	1 kb
Wireless technology	IEEE 802.11 DCF
Maximum UAV speed	5∼25 m/s
Modulation	Modulation_bpsk
Fading model	Nakagami_m
Carrier frequency	30 GHZ
Carrier wavelength	0.01 m
Electromagnetic wave speed	3 × $10^{8}$ m/s
Traffic type	Constant bit rate (CBR)
μ	0.6
ν	0.4
δ	−0.5
φ	−0.5
Buffer size	32 KB
Experience relay pool size	2000
Adam learning rate	0.001
τ	0.05
γ	0.99

## Data Availability

Not applicable.
